# Bacterial-fungal interactions under agricultural settings: from physical to chemical interactions

**DOI:** 10.1007/s44154-022-00046-1

**Published:** 2022-06-01

**Authors:** Yaqi Zhou, Hongkai Wang, Sunde Xu, Kai Liu, Hao Qi, Mengcen Wang, Xiaoyulong Chen, Gabriele Berg, Zhonghua Ma, Tomislav Cernava, Yun Chen

**Affiliations:** 1 State Key Laboratory of Rice Biology, and Key Laboratory of Molecular Biology of Crop Pathogens and Insects, Institute of Biotechnology, Zhejiang University, 866 Yuhangtang Road, Hangzhou, 310058 China; 2 Key Laboratory of Molecular Biology of Crop Pathogens and Insects, Ministry of Agriculture, Institute of Pesticide and Environmental Toxicology, Zhejiang University, Hangzhou, China; 3 Key Laboratory of Green Pesticide and Agricultural Bioengineering, Ministry of Education, Guizhou University, Guiyang, 550025 China; 4 Institute of Environmental Biotechnology, Graz University of Technology, 8010 Graz, Austria; 5 Leibniz-Institute for Agricultural Engineering and Bioeconomy, Potsdam, Germany; 6 University of Potsdam, Potsdam, Germany

**Keywords:** Bacterial-fungal interactions, Biological control, Synthetic communities, Secondary metabolites

## Abstract

Bacteria and fungi are dominant members of environmental microbiomes. Various bacterial-fungal interactions (BFIs) and their mutual regulation are important factors for ecosystem functioning and health. Such interactions can be highly dynamic, and often require spatiotemporally resolved assessments to understand the interplay which ranges from antagonism to mutualism. Many of these interactions are still poorly understood, especially in terms of the underlying chemical and molecular interplay, which is crucial for inter-kingdom communication and interference. BFIs are highly relevant under agricultural settings; they can be determinative for crop health. Advancing our knowledge related to mechanisms underpinning the interactions between bacteria and fungi will provide an extended basis for biological control of pests and pathogens in agriculture. Moreover, it will facilitate a better understanding of complex microbial community networks that commonly occur in nature. This will allow us to determine factors that are crucial for community assembly under different environmental conditions and pave the way for constructing synthetic communities for various biotechnological applications. Here, we summarize the current advances in the field of BFIs with an emphasis on agriculture.

## Introduction

In nature, microorganisms often form complex communities, also known as the microbiome (Berg et al. 2020). Within microbiomes, members of the microbiota, which include bacteria, archaea, fungi, algae and protists, are connected by various types of intra-kingdom as well as inter-kingdom interactions (Berg et al. 2020; Braga et al. 2016). In terms of inter-kingdom interactions, bacterial-fungal interactions (BFIs) were often studied in various fields of microbial ecology (Frey-Klett et al. 2011). Bacteria and fungi, which are both essential for the functioning of most ecosystems, are also crucial for health and diseases in various organisms (Frey-Klett et al. 2011). It is commonly observed that phytopathogenic fungi occur within the microbiota of healthy plants (Manzotti et al. 2020). Inference analyses from microbial networks indicate that such fungi are naturally suppressed by other members of the microbiota, mostly bacteria (Wassermann et al. 2019). This and other observations led to the introduction of the term soterobiont which specifically describes disease-preventing microorganisms within the host-associated microbiota (Cernava and Berg, 2022). Recent results showed that host- and bacterium-encoded functions act in concert to balance BFIs in Arabidopsis roots, thereby promoting plant health and maintaining growth-promoting activities of multi-kingdom microbial communities (Wolinska et al. 2021). Under environmental conditions, bacteria and fungi are involved in numerous interactions ranging from antagonism to mutualism, affecting the growth, reproduction, transport/movement, nutrition, stress resistance and pathogenicity of the involved partners to varying degrees (Deveau et al. 2018). These interactions have specific implications for host health if they occur within plant or animal microbiota and can be determinative for health and disease (Berg et al. 2021).

**Fig. 1:**
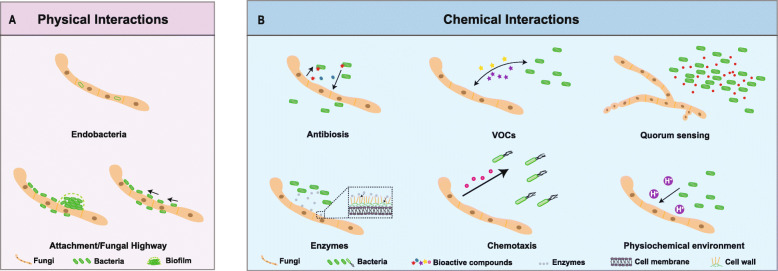
Modes of bacterial-fungal interactions. Bacteria and fungi interact with each other in various ways and can influence each other’s survival or virulence. The consequences of these interactions can be beneficial or harmful for the involved partners. Generally, these interactions can be divided into physical and chemical interactions. **A** Physical interactions include bacterial endosymbionts of fungi or attachment to the fungal surface. **B** Diverse bacteria or fungi produce small molecules (antibiosis, volatile organic compounds, quorum sensing molecule etc.) to affect the partners, including morphology, growth, reproduction, transport/movement, nutrition, stress resistance and pathogenicity

In this review, we differentiate between physical interactions and chemical interactions between fungi and bacteria (Fig. 1). Physical interactions are often considered as the simplest interactions between bacteria and fungi. By using fungi as a scaffold, bacteria can be located inside or outside the fungus. This interaction depends not only on the morphology of the fungus, but also on the surface molecules and secretory factors of the involved microbes (Steffan et al. 2020). When bacteria and fungi are in direct contact, they often exchange or react to each other’s metabolites, and in some special cases, bacteria can invade the interior of the fungus and live there as endophytes (Mosse 1970). Chemical interactions affect interacting partners primarily through the production of secondary metabolites (SMs). Here, antibacterial and antifungal compounds play an important role because they are often involved in antagonistic interactions (Hutchings et al. 2019). A specific sub-class is constituted by volatile organic compounds (VOCs) which are constituted by highly diversified secondary metabolites of low molecular weight (Schmidt et al. 2015). Quorum-sensing (QS) molecules are another group of commonly small molecules that microbes release to communicate with other partners and to determine when a critical mass is reached (Zhao et al. 2017). Physical interactions (planktonic, mixed biofilm, intrahyphal colonization), chemical interactions (direct or indirect), adaptations to environmental conditions, and/or host colonization are mostly preceded by successful molecular communication of the involved partners. Therefore, small molecules involved in QS and VOCs signaling are increasingly coming into the spotlight of BFIs research.

Here, we review various BFIs relevant for agriculture that have been reported during the last years. In addition, we also highlight recent results from a few representative clinical as well as other studies targeting BFIs in order to draw parallels and to identify specific overlaps with agricultural research. By understanding prevalent inter-kingdom interactions within complex microbial communities, we can identify crucial drivers of their assembly which will allow us to engineer beneficial microbiomes in the future to control diseases more efficiently.

## Physical interactions between bacteria and fungi

### Bacterial endosymbionts of fungi

Endofungal bacteria or endobacteria are bacterial symbionts that inhabit inner compartments of fungal mycelia (Mosse 1970) (Table 1). To date, endobacteria have been detected in fungi with various lifestyles and a wide range of taxonomic origins, ranging from various ascomycota (Deveau et al. 2018), basidiomycota (Ruiz-Herrera et al. 2015) as well as saprotrophic and symbiotic fungi of the Mucoromycota (Desiro et al. 2018). Most of the discovered endobacteria have lost the ability to live independently and have undergone extensive genomic reduction, completing their life cycle using the fungal cytoplasm as a place for generations to live (Deveau et al. 2018; Pawlowska et al. 2018). In a recent study, the genome of the culturable endobacterium *Mycoavidus* sp. strain B2-EB was compared to genome sequences of other endobacteria, and showed high genomic integrity revealing the minimal genomic characteristics required for fungal lifestyle and artificial cultivation conditions (Guo et al. 2020). Several classic examples of endobacteria are found in the family of Burkholderiaceae, which can live in microspores of *Rhizopus* spp. This fungus is an important plant pathogen that can together with its symbiont cause rice seedling blight. It produces rhizoxin, a biotoxin with antimitotic properties required for its pathogenicity and previously incorrectly identified as a mycotoxin (Iwasaki et al. 1984). Later, Partida-Martinez and Hertweck (2005) found that rhizoxin was synthesized by the endobacterium *Burkholderia* rather than by *R. microspora*. Scherlach (2012) further showed that the synthesis enzymes of 2, 3-oxirane ring, the precursor of rhizoxin, came from *R. microspora*. Therefore, both play an important role in the biosynthesis of rhizoxin. It was also discovered that in the absence of endobacteria, *R. microsporum* could not reproduce and the production of spores could only be restored after the re-introduction of endobacteria (Partida-Martinez et al. 2007).

**Table 1:** Physical bacterial-fungal interactions in agriculture

Bacteria	Fungi	Functions	Reference
**Endobacteria**			
*B. rhizoxinica*	*R. microsporus*	Rhizoxin synthesized by *B. rhizoxinica* and *B. rhizoxinica* controls the growth of fungal spores	(Lackner et al. 2011)
*Candidatus* Glomeribacter gigasporarum	*Mortierella*	*Candidatus* Glomeribacter gigasporarum protects fungi from nematodes	(Buttner et al. 2021)
*Mollicutes*/*Mycoplasma*-related endosymbionts associated	Glomeromycotina, Mucoromycotina	Endobacteria impose some fitness costs to their fungal host	(Desiro et al. 2018)
*Mycoavidus cysteinexigens*	*Mortierella elongata*	*M. cysteinexigens* is a parasite of *M. elongata*	(Pawlowska et al. 2018)
*Rhizobium radiobacter*	*Serendipita indica*	Symbiosis promotes plant growth and improves systemic resistance	(Guo et al. 2017)
**Attachment**			
*B. subtilis*	*A. niger,* *A. bisporus*	Extracellular polysaccharides, TasA and Spo0A of *B. subtilis* play an important role in bacterial attachment to fungi	(Kjeldgaard et al. 2019)
*B. subtilis*	*Ceratocystis fimbriata*	Enhanced spoVF operon increases attachment to fungi and biocontrol ability of *B. subtilis*	(Wang et al. 2021)
*B. subtilis*	*A. nidulans*	*B. subtilis* reaches the mycelial edge and provides thiamine to the growing hyphae	(Abeysinghe et al. 2020)
*P. fluorescens, B. cereus*, *P. peoriae*	*Glomus* sp. MUCL 43205, *Glomus intraradices* MUCL 43194	Fungal hyphae affect bacterial attachment	(Toljander et al. 2006)
*P. fluorescens*	*Gigaspora margarita*	*P. fluorescens* adhere to spores and hyphae of AM fungi germinated under sterile conditions	(Bianciotto et al., 1996b)
*P. putida*	*Pythium ultimum*	*P. putida* can migrate in the presence of fungi	(Furuno et al. 2010)
*S. marcescens*	*Rhizopus oryzae*	*S. marcescens* with defective pilus expression can migrate more quickly along fungi	(Hover et al. 2016)
*S. proteamaculans*	*Mucor*	*S. proteamaculans* disperses on fungal networks and can shape microbial community structure	(Zhang et al. 2018)

A second widely studied example of endobacteria is found in arbuscular mycorrhizal fungi (AMF) from the Gigasporaceae family which can host a *Burkholderia*-related microbe (Bianciotto et al., 1996a) that was named *Candidatus* Glomeribacter gigasporarum (*Ca*gg). *Ca*gg is vertically transmitted and cannot be cultivated. Although this is not necessary for *Gigaspora* survival, *Ca*gg can enhance the bioenergetic capacity of the fungus, increase ATP production, and induce mechanisms to detoxify reactive oxygen species (Salvioli et al. 2016). AMF can contain two groups of endobacteria, namely, *Mollicutes*-related endobacteria (MRE) and the aforementioned *Ca*gg. The distribution patterns and coexistence of MRE and *Ca*gg in different hosts were extensively studied, and it was found that a single AMF host can harbor both types of endobacteria; MRE is more widely associated with AMF, more abundant, and variable than *Ca*gg (Desiro et al. 2014).

At present, the process and the required molecular mechanisms of bacteria that can enter fungal hyphae are not entirely understood. It is known that bacterial entry into fungal hosts requires inhibition of the host cell’s defense systems and that protein secretion systems (T1SS-T7SS) are involved. Moebius et al. (2014) discovered that the process of bacteria invading fungal hyphae involves secretion of chitinolytic enzymes. In combination with the type II secretion systems (T2SS) (Moebius et al. 2014), secreted chitinases are the major mechanisms enabling bacterial invasion of fungi. Chitinases as well as other effector proteins can be secreted via T2SS and induce a local dissolution of the fungal cell wall. This enables bacteria to enter and colonize fungal cells and induce sporulation. However, there are also other strategies that can be employed by various bacteria. It was shown that T6SS was involved in the invasion process when the interaction between the endobacterium *Serratia marcescens* D1 and *Mucor irregularis* SS7 was studied (Hazarika et al. 2020). Microbes can be vertically transmitted from one generation to the other in many organisms; however, there is still little known about endofungal bacteria.

### Bacterial attachment to fungal surfaces

Bacteria can also attach to the surface of fungi where they can engage in various interactions (Table 1). The mechanisms of bacterial attachment to the surface of fungi have been investigated extensively both in medicine and agriculture. It was shown that surface factors and secretory factors are crucial for this specific interaction between fungi and bacteria (Steffan et al. 2020).

*Candida albicans* is a polymorphic fungus that is also an opportunistic human pathogen that can cause common mucosal infections as well as serious life-threatening systemic infections in immuno-compromised patients suffering from HIV-infection or such that underwent a transplantation (Odds 1988). It was shown to co-occur with highly diverse bacteria (*Staphylococcus, Streptococcus, and Pseudomonas*) in different parts of the human body where it forms mixed-species biofilms (Ovchinnikova et al. 2012). In order to enhance survival and growth, microorganisms within mixed-species communities can be involved in various interactions. *C. albicans* and *Pseudomonas aeruginosa* are commonly isolated together in catheter-related infections or infections in patients with cystic fibrosis and burn wounds (Falleiros et al. 2008). Detailed analyses showed that the outermost mannoprotein layer on hyphal surfaces creates favorable acid-base conditions for adhesion, allowing *P. aeruginosa* to attach to the fungal surface. In contrast, the absence of these proteins causes unfavorable conditions, preventing adhesion of *P. aeruginosa* (Ovchinnikova et al. 2012)*.*

In the context of agriculture, microbial attachment factors, secretion factors and surface factors have been widely reported. The physicochemical properties of fungal hyphae can substantially affect bacterial attachment. Attachment properties can also differ between the involved species and strains. In one study, *Pseudomonas fluorescens* was shown to attach to both living and non-living mycelia, whil*e Bacillus cereus* and *Paenibacillus peoriae* showed better attachment to non-living mycelia and *Paenibacillus brasilensis* only attached to living mycelia (Toljander et al. 2006). Hover et al. (2016) found that *Serratia marcescens* with defective pilus expression can migrate more quickly, suggesting that pilus adhesion hinders migration, perhaps by forming a dense bacterial biofilm or adhering to the mycelium tightly. Hence, a looser attachment between bacteria and fungi is conducive to bacterial migration. Moreover, the formation of biofilms can enhance successful attachment of bacteria to fungi. *Bacillus subtilis* can form biofilms on the hyphae of *Aspergillus niger* and *Agaricus bisporus*. Here, the formation of biofilms depends on extracellular polysaccharides (EPS) and the main biofilm matrix component (TasA, amyloid fibers) secreted by bacteria, which are regulated by Spo0A. If any of these components are knocked out, *B. subtilis* was shown to no longer form biofilms and it was not able anymore to attach to fungal surfaces. The addition of matrix components to mutants restored biofilm formation (Kjeldgaard et al. 2019).

Some bacteria can use fungal hyphae as a so-called “fungal highway”, giving them a distinct advantage when spreading in various environments. Most bacteria are directed via chemotaxis to fungi (Steffan et al. 2020). *Serratia* spp. were shown to move along fungal hyphae where they can also engage in antagonistic interactions. It was also shown that migration is restricted to zygomycetes and several basidiomycete species; it may be related to the structural properties of the fungi (Hover et al. 2016). The migration of bacteria along fungi can also be employed as a strategy to colonize new micro-environments that would be otherwise inaccessible. *Serratia proteamaculans* and other motile cheese rind bacteria disperse on fungal networks using the liquid layers formed on fungal hyphae (Zhang et al. 2018). For *B. subtilis* it was shown that it can move fast along *Aspergillus nidulans* hyphae while being involved in mutualistic interactions by the provision of thiamine to the growing hyphae (Abeysinghe et al. 2020). This results in growth promotion of the fungus and is indicative of a symbiotic relationship.

## Chemical interactions between bacteria and fungi

### Interactions via antibiosis

Antibiotics are secondary metabolites that are often isolated from microorganisms and have antibacterial, antitumor or antiviral activities which are applicable in medicine (Berdy 2005). In nature, the production of antibiotics, and more generally antimicrobial compounds, by soil or plant-associated bacteria and fungi provides advantages for their own growth and makes them more persistent in competitive environments (Scherlach et al. 2013). Especially in nutrient-limited environments, antibiotics are assumed to account for the most effective mode of action against competitors because a significant competitive advantage is given to microbes via such metabolites (Raaijmakers and Mazzola 2012). They are often strong effectors and can induce various responses in target organisms, including changes in transcription, virulence, motility, and biofilm formation (Romero et al. 2011). The use of antimicrobial compounds for biological control in agriculture has been thoroughly studied (Table 2) and showed many parallels to classical drug discovery.

**Table 2:** Bacterial-fungal interactions via antimicrobial compounds in agriculture

Producer	Receiver	Compounds	Functions	Reference
*B. subtilis,* *B. amyloliquefaciens*	*B. cinerea,* *Penicilium digitatum, Fusarium* spp.*,* *Aspergillus phoenicis,* *Bipolaris sorokiniana,* *Sclerotinia sclerotiorum,* *Magnaporthe grisea,* *Verticillium dahliae*	Fengycin	Antifungal activity and effect on fungal cell membranes	(Liu et al. 2005)
*B. subtilis,* *B. amyloliquefaciens,* *B. circulans*	*F. oxysporum,* *Gloeosporium gloeosporioides, Rhizoctonia solani, Phomopsis* spp.*,* *Alternaria citri*	Iturin A	Antifungal activity and effect on fungal cell membranes	(Cazorla et al. 2007)
*B. subtilis,* *B. vallismortis,* *B. mojavensis,* *B. amyloliquefaciens*	*F. oxysporum, Alternaria alternata,* *R. solani, Cryphonectria parasitica,* *A. flavus, Colletotrichum gloeosporioides*	Bacillomycin D	Antifungal activity	(Zhao et al. 2014)
*B. subtilis,* *B. licheniformis,* *B. amyloliquefaciens*	*Pestalotiopsis eugeniae, M. grisea,* *F. verticillioides, S. sclerotiorum,*	Surfactin	Antifungal activity and destruction of fungal cell membranes	(Bais et al. 2004)
*Burkholderia cepacia*	*A. alternata, A. niger,* *F. culmorum, F. graminearum,* *F. oxysporum, R. solani*	Pyrrolnitrin, benzoic acid, phenylacetic acid	Antifungal activity	(Jung et al. 2018)
*Burkholderia ambifaria*	*P. ultimum*	Cepacin A	Antifungal activity	(Coenye 2019)
*Burkholderia* sp. *HD05*	*Saprolegnia* sp.	2-pyrrolidone-5-carboxylic acid	Antifungal activity	(Zhang et al. 2019)
*Burkholderia* sp. MS455	*A. flavus*	Occidiofungin	Antifungal activity	(Jia et al. 2021)
*Brevibacillus brevis*	*B. cinerea,* *F. oxysporum* f. sp. *lycopersici*	Gramicidin S, polymyxin B	Antifungal activity and destruction of fungal cell membranes	(Chandel et al. 2010)
*E. nigrum*	*X. albilineans*	Flavipin	Antibacterial activity	(Favaro et al. 2012)
*F. fujikuroi*	*R. solanacearum*	Bikaverin	Antibacterial activity	(Spraker et al. 2018)
*L. enzymogenes* OH11	*R. solani, S. scletotiorum*	Heat stable antifungal factor	Antifungal activity and inhibited ceramide synthase	(Qian et al., 2009)
*P. chlororaphis,* *P. aeruginosa*	*F. oxysporum, R. solani*	Phenazine 1-carboxylic acid	Antifungal activity	(Trung et al. 2020)
*P. chlororaphis,* *P. aeruginosa*	*F. oxysporum, F. graminearum*	Phenazine 1-carboxamide	Antifungal activity and inhibited gene expression	(Chen et al., 2018)
*P. fluorescens,* *P. aurantiaca*	*Gaeumannomyces gramini* var*. tritici,* *F. oxysporum,*	2,4 Diacetylphloroglucinol	Antifungal activity	(Deng et al. 2015)
*P. chlororaphis*	*G. graminis* var*. tritici*	2-Hydroxyphenazine, 2-hydroxy-phenazine-1-carboxylic acid	Antifungal activity	(Liu et al. 2016)
*P. fluorescens* *P. chlororaphis*	*R. solani, F. graminearum,* *B. cinerea*	Pyrrolnitrin	Antifungal activity and inhibited cellular respiration	(Huang et al. 2018)
*P. chlororaphis*	*B. cinerea, Phytophthora ramorum*	Rhizoxin	Antifungal activity	(Loper et al. 2008)
*S. hygroscopicus*	*F. graminearum*	Rapamycin	Antifungal activity and affected gene acetylation	(Wang et al., 2021)
*Streptomyces* sp. isolate UPMRS4	*Pyricularia oryzae*	Ergotamine, Amicomacin, Fungichromin, Rapamycin, N-Acetyl-D, L-phenylalanine	Antifungal activity	(Awla et al. 2016)
*Streptomyces palmae*	*Ganoderma boninense*	Actinopyrone A, Anguinomycin A, Leptomycin A	Antifungal activity	(Sujarit et al. 2020)
*Streptomyces griseus*	*Phytophthora capsici*	1H-pyrrole-2-carboxylic acid	Antifungal activity	(Nguyen et al. 2015)
*Streptomyces* sp. *CEN26*	*Alternaria brassicicola*	2,5-Bis (hydroxymethyl) furan monoacetate	Antifungal activity	(Phuakjaiphaeo et al. 2016)
*Streptomyces* sp. *M4*	*Alternaria* spp., *Fusarium* spp., *Colletotrichum* spp.	Salvianolic acid B	Antifungal activity	(Sharma and Manhas 2020)
*Pantoea ananatis* 4G-9	*Mycosphaerella musicola*	Indole derivative	Antifungal activity	(Aman and Rai, 2016)
*Pantoea jilinensis*	*B. cinerea*	1,2-benzenedicarboxylic acid bis(2-methylpropyl) ester, 2,4-di-tert-butylphenol, 2,6,10,14-tetramethyl pentadecane, 1,3-dimethyl naphthalene	Antifungal activity	(Zheng et al. 2021)
*P. polymyxa*	*F. oxysporum,* *R. solani,* *F. graminearum*	Fusaricidin A	Antifungal activity and effects on cell wall	(Liu et al. 2011)

Various antibiotics often play dual roles, ranging from antimicrobial activity to inducing changes in morphological development or gene expression in the receiving organism. For example, phenazine-derived compounds that are produced by *Pseudomonas* spp. are often regarded as microbial toxins that inhibit the growth of several fungal species. However, it has been also found that lower doses of phenazines can induce sporulation in *Aspergillus fumigatus* (Zheng et al. 2015)*.* In another example, the lipopeptide ralsolamycin produced by the plant-pathogenic bacterium *Ralstonia solanacearum* can induce bikaverin biosynthesis in the plant-pathogenic fungus *Fusarium fujikuroi* which then leads to growth suppression of *R. solanacearum* (Spraker et al. 2018)*.*

By influencing each other’s growth some plant-associated bacteria and fungi can improve plant health, growth, and fitness (Carrasco and Preston 2020). For example, the plant-endophytic bacterium *B. subtilis* produces antifungal lipopeptides (e.g., surfactin) that suppress phytopathogenic pathogenic *Fusarium* spp. (Gond et al. 2015). On the other hand, the antibacterial metabolite flavipin produced by the plant-endophytic fungus *Epicoccum nigrum* is effective against plant-pathogenic bacteria, i.e., *Xanthomomas albilineans* (Favaro et al. 2012)*.*

Despite a high number of studies that focused on antimicrobial compounds in BFIs, we still do not know their exact function as well as expression under natural conditions. In addition to competition, signaling and change of mutation frequency were suggested as functions (Linares et al. 2006). Targeted transcriptomics studies under in vitro conditions are suggested to better understand their role in vivo (Alavi et al. 2013).

### Interactions via volatile organic compounds

Both bacteria and fungi can produce a wide range of volatile organic compounds (VOCs) as a part of their secondary metabolism. In recent years, VOCs came into the spotlight due to their role as communication molecules (Schmidt et al. 2015). Due to their unique nature (low molecular mass, low boiling point and often lipophilic properties), VOCs can evaporate and diffuse through air and water-filled voids in soil, acting as chemical mediators for long-distance microbial interactions (Schmidt et al. 2015). VOCs can cause various physiological reactions of bacteria or fungi, and affect the growth and health of plants and animals (Netzker et al. 2020). Bacterial volatiles are mainly alkenes, alcohols, ketones, terpenes, benzenoids, pyrazines, organic acids and esters, whereas fungal volatiles are mainly alcohols, benzenoids, aldehydes, alkenes, acids, esters and ketones (Piechulla and Degenhardt 2014). In this review, a briefly summary of bacterial-fungal interactions based on VOCs is presented (Table 3). A more detailed overview on how bacterial VOCs affect fungi was recently summarized by Netzker and colleagues (Netzker et al. 2020).

**Table 3:** Bacterial-fungal interactions via VOCs in agriculture

Producer	Receiver	Compounds	Functions	Reference
*A. faecalis*	*A. flavus*	Dimethyl disulfide (DMDS), methyl isovalerate (MI)	Antifungal activity and affected gene expression in aflatoxin biosynthesis	(Gong et al. 2019)
*B. amyloliquefaciens*	*F. oxysporum* f. sp. *cubense*	Benzothiazoles phenol, 2,3,6-trimethyl-phenol	Antifungal activity	(Yuan et al. 2012)
*B. subtilis* M29	*B. cinerea*	1-butanol, acetic acid butyl ester, 1-heptylene-4-alcohol, 3-methyl-3-hexanol	Antifungal activity	(Mu et al. 2017)
*B. amyloliquefaciens,* *B. velezensis* UQ156*, Acinetobacter* sp. UQ202	*P. capsici*	Aldehydes, alcohols, esters, carboxylic acids, ketones	Antifungal activity and promoted plant growth	(Syed-Ab-Rahman et al. 2019)
*B. subtilis* C9*,* *Pseudomonas trivialis* 3Re2–7*,* *Serratia odorifera* 4Rx13	*R. solani*	Isomer of acetylbuanediol, benzyloxybenzonitrile, dimethyl trisulfide	Antifungal activity and promoted plant growth	(Islam et al. 2012)
*Burkholderia gladioli* pv. *agaricicola*	*B. cinerea, A. flavus, A. niger,* *Penicillium digitatum, Penicillium expansum, S. sclerotiorum,* *Phytophthora cactorum*	1-methyl-4-(1-methylethenyl)-cyclohexene	Antifungal activity	(Barka et al. 2002)
*F. culmorum*	*S. plymuthica, Collimonas pratensis*	Terpene	Inducement of bacterial movement	(Schmidt et al. 2017)
*P. ostreatus*	*B. cereus,* *B. subtilis*	1-octen-3-ol, 3-octanol, octanol, 3-octanone, 2-octanone	Antibacterial activity	(Pauliuc and Botau 2013)
*P. polymyxa,* *V. longisporum*	*V. longisporum,* *P. polymyxa*	1-butanol (fungi), durenol (bacteria)	Synthesis of durenol is downregulated, and the production (1-butanol) is upregulated	(Rybakova et al. 2017)
*Pseudomonas* spp.*,Micromonospora* spp.	*P. ostreatus,* *P. eryngii*	E)-12-methyltridec-3-enenitrile	Antifungal activity	(Lo et al. 2015)
*Streptomyces* spp.	*B. cinerea*	3-carene 2,5-dione, geosmin, beta-cubebene Phenol, 2-(1,1 dimethylethyl)-6-methyl-	Antifungal activity	(Ayed et al. 2021)
*T. atroviride*	*P. fluorescens*	Unknown	Increased expression of 2,4-diacetylphloroglucinol	(Lutz et al. 2004)

Volatiles produced by bacteria are commonly inhibiting the germination of fungal spores and the growth of mycelia (Herrington et al. 1987) or changing the morphology, enzyme activity and gene expression of fungi (Kai et al. 2009). Bacteria were so far shown to release larger and more diverse volatile compounds than fungi. These volatiles can have strong bioactive effects. In *Fusarium oxysporum*, hyphae-associated bacteria were shown to produce the volatile sesquiterpene caryophyllene, which repressed the expression of two virulence genes in the pathogenic fungus. In the absence of endobacteria caryophyllene was not detected and *F. oxysporum* became pathogenic (Minerdi et al. 2008). Beyond inhibiting fungal growth, bacterial VOCs can also be used to reduce mycotoxin production. Aflatoxin is produced by *Aspergillus flavus* and seriously affects human and livestock health. *Alcaligenes faecalis* N1–4 isolated from the tea rhizosphere could produce two antifungal volatiles including dimethyl disulfide (DMDS) and methyl isovalerate (MI), which significantly inhibit the mycelia growth and gene expression in aflatoxin biosynthesis (Gong et al. 2019).

Fungal VOCs also play an important role in long-distance bacterial-fungal interactions, and thus many studies have focused on the effects of fungal VOCs on bacteria or the assembly of bacterial communities. VOCs released by *Trichoderma atroviride* increased expression of biocontrol genes encoding 2, 4-diacetylphloroglucinol by the antagonistic bacterium *P. fluorescens* (Lutz et al. 2004)*.* The oyster mushroom *Pleurotus ostreatus* produces volatiles with inhibitory effects on *B. cereus* and *B. subtilis* (Pauliuc and Botau 2013)*.* In another study, it was shown that the plant-pathogenic fungus *Fusarium culmorum* produces a unique mixture of VOCs, consisting primarily of terpenes. When exposed to the VOCs emitted by this fungus, the rhizobacterium *Serratia plymuthica* PRI-2C responded with an induction of motility (Schmidt et al. 2017). In addition to inhibiting individual microorganisms, VOCs also have an impact on microbiome assembly. In a study focusing on cheese rind, the total abundance of *Vibrio* sp. increased when exposed to fungal VOCs (Cosetta et al. 2020).

The abovementioned studies mainly focus on the influence of VOCs from bacteria/fungi on one interaction partner. However, distinct studies were conducted during the last years where reciprocal interactions were assessed. *Paenibacillus polymyxa* emits volatiles that inhibit the growth of the wilt-causing fungus *Verticillium longisporum* and leads to the simultaneous downregulation of fungal metabolic activity as well as activation of antimicrobial compound production (e.g., isobutanol, 2-phenylethanol) (Rybakova et al. 2017). At the same time, exposure of *P. polymyxa* to fungal volatiles results in a general upregulation of metabolic activity. A two-way volatile interaction has been also described for the plant-pathogenic bacterium *R. solanacearum* and the plant-pathogenic fungus *A. flavus*. Volatiles produced by *R. solanacearum* resulted in both decreased conidiation and increased aflatoxin production by *A. flavus*. Conversely, exposure of *R. solanacearum* to fungal volatiles led to a decreased growth rate, reduced melanin production, and increased extracellular polysaccharide production (Spraker et al. 2014).

### Interactions via enzymes

Parasitism and lysis constitute direct interactions between microorganisms (Whipps 2001); i.e. bacteria can secrete cell wall-degrading enzymes such as chitinases, β-1,3-glucanases, proteases or cellulases in combination with secondary metabolites for killing and invading fungi (Liu et al. 2007). Chitinolytic microorganisms of the genera *Bacillus, Serratia* and *Trichoderma* are powerful biocontrol agents (Berg et al. 2002). Most of the current research on extracellular lytic enzymes in *Lysobacter* focuses on chitinase and β-1,3-glucanases, which are particularly important in microbial antagonistic activity and biological control (Palumbo et al. 2005). Interestingly, the risk of resistance formation by a plant pathogen is low when subjected to this type of antagonism, though pathogens are able to evolve resting structures like endospores, chlamydospores and melanized sclerotia, or suppress the synthesis of antagonistic enzymes for stemming hyperparasitic interactions (Bardin et al. 2015).

### Communication via quorum sensing between bacteria and fungi

Quorum sensing (QS) is a microbial communication system based on auto-inducers to various functions within communities. QS is mostly known in bacteria where it provides the means of sensing and regulating its population density to coordinate the overall activity. Analogous functions have been also described in fungi where QS was shown to be also involved in processes such as morphogenesis, germination, apoptosis, pathogenicity and biofilm development (Wongsuk et al. 2016). QS plays a major role in BFIs. For example, *N*-acyl-L-homoserine lactone (AHL), a common bacterial QS molecule, not only controls the synthesis of compounds active against other organisms but they are also recognized by various eukaryotes, including animal cells, plants, seaweed and fungi (Dudler and Eberl 2006). On the other hand, some studies show that fungi can interfere with bacterial QS through their own QS systems (Cugini et al. 2008) as well as by producing AHL antagonists (Rasmussen et al. 2005).

The *C. albicans* QS molecule farnesol leads to the downregulation of *Pseudomonas* quinolone signal (PQS) in *P. aeruginosa* and, consequently, of pyocyanin production (Cugini et al. 2007). On the other hand, 3-oxo-C12 HSLs produced by *P. aeruginosa* inhibits the transformation of *C. albicans* from yeast to mycelia (Hogan et al. 2004). Our understanding related to the roles of QS signaling in BFIs is still relatively shallow, although it is known that QS is present in many environmental bacteria. There is some evidence that mycorrhizal fungi can degrade QS molecules (Frey-Klett et al. 2011). It was also shown that in the process of antagonistic interactions with *Setophoma terrestris*, a soil-associated *B. subtilis* strain developed heritable phenotypic variation and metabolic transfer through ComQXPA QS system mutation (Albarracin et al. 2020).

### Bacterial chemotaxis in bacterial-fungal interactions

Many motile microorganisms will move towards or away from a concentration gradient of various chemical substances. This phenomenon is known as chemotaxis. Various studies have demonstrated the occurrence of chemotaxis in BFIs. For example, harmful and beneficial *Pseudomonas* can show a tendency to migrate towards exudates of fungal hyphae (Grewal and Rainey 1991). The fungus *F. oxysporum* f. sp. *radicis-lycopersici* can release fusaric acid as a chemotactic signal to attract *P. fluorescens* WCS365 (de Weert et al. 2004). In addition, oxalic acid was shown to be commonly secreted by soil fungi and to play an important role in the recruitment of various bacteria from the local environment (Rudnick et al. 2015). With the help of flagella, bacteria can sense pathogens or environmental signals through the chemosensory motility system, allowing them to migrate to an environment more conducive to their survival. However, there are also alternative systems. The biocontrol bacterium *Lysobacter enzymogenes* OH11, which lacks flagella and secrets a diffusible antibiotic heat-stable antifungal factor (HSAF), relies on the Wsp chemosensory system to enhance the secretion of HSAF (Xu et al. 2021).

### Effects of physiochemical factors on bacterial-fungal interactions

The interactions between bacteria and fungi can be altered by changing the physicochemical environment; one of the most common influencing parameters is the local pH. Bacteria generally prefer alkaline environments, while fungi generally prefer acidic environments which makes them more resistant to such environments than bacteria (Fierer and Jackson 2006). Thus, pH changes can promote or inhibit interactions between two specific microorganisms and affect the composition of whole microbial communities that are present in a specific environment. On cheese surfaces, lactic acid metabolism and the production of alkaline metabolites such as ammonia can cause deacidification. This change favors the growth of less-acid-tolerant bacteria; they are essential for ripeness, flavor and quality of the cheese (Corsetti et al. 2001). Secretion of gluconic acid (GlcA) by *Rahnella aquatilis* in the plant rhizosphere leads to acidification and counteracts *F. oxysporum*-induced alkalinization, a known virulence mechanism, thereby preventing fungal infection (Palmieri et al. 2020). In addition to affecting the growth of microorganisms, it also affects the synthesis of microbial secondary metabolites. Under acidic conditions, the synthesis rate of aflatoxin produced by *Aspergillus* spp. is higher than under alkaline growth conditions, while alkaline medium increases the production of penicillin by *A. nidulans* (Penalva and Arst 2002)*.*

### Activation of biosynthetic gene clusters

Both bacteria and fungi produce a large number of secondary metabolites, but they can also contain a large number of silent biosynthetic gene clusters. The vast majority of secondary metabolite biosynthetic gene clusters (BGCs) are poorly expressed under laboratory conditions. Therefore, their corresponding metabolites remain largely unknown (Zhang and Hindra 2019). It has been widely reported that bacterial and fungal co-cultivation can activate the expression of specific BGCs and stimulate the production of novel metabolites with various activities. It was also shown that co-cultivation can be a promising strategy for the discovery of new antibacterial agents.

*Bacillus*, *Pseudomonas*, and *Streptomyces* species were reported to be the most commonly found bacteria in soil or the rhizosphere and play the most important role as fungal partners. Studies (Ola et al. 2013) found an up to 78-fold increase in the accumulation of constitutively present fungal products such as the lipopeptide fusaristatin A (Cueto et al. 2001) in the presence of *B. subtilis* compared to axenic cultures of *Fusarium tricinctum*. *Streptomyces* is the largest genus in the Actinomycetes order and is the source of a wide range of bioactive compounds. When *Streptomyces rapamycinicus* and *A. fumigatus* ATCC 46645 were co-cultured, *S. rapamycinicus* was shown to induce the expression of the Fgn gene cluster to produce fumigermin. Fumigermin inhibits germination of spores in *S. rapamycinicus* (Stroe et al. 2020)*.* It was also found that various combinations of bacteria and fungi in co-cultures can induce the production of different types of compounds. For example, a co-culture of *B. subtilis* and the endophytic fungus *F. tricinctum* induced the fungus to produce lateropyrone and other compounds (Ola et al. 2013). However, when co-cultured with another endophytic fungus, *Chaetomium* sp., the fungus produced serkydayn (Akone et al. 2016).

### Epigenetic modification by bacteria

Various studies showed that bacteria can trigger changes in fungal epigenetic modification. A growing body of evidence demonstrated that acetylation plays an essential role in mediating various bacterial-fungal interactions. Antagonistic compounds secreted by bacteria can interfere with fungal chromatin remodeling and the transcription of target genes. For example, the antifungal compound phenazine-1-carboxamide (PCN) produced by *Pseudomonas chlororaphis* ZJU60 can directly affect the activity of the fungal histone acetyltransferase FgGcn5 in *Fusarium graminearum*, subsequently leading to histone deacetylation of H2BK11, H3K14, H3K18 and H3K27 and inhibiting fungal growth, virulence and mycotoxin biosynthesis (Chen et al. 2018). Rapamycin produced by *Streptomyces hygroscopicus* acts on the TOR signaling pathway to promote the degradation of FgGcn5 through the 26S proteasome, thereby reducing the acetylation level of Atg8 and promoting autophagy. (Wang et al. 2021). In another interaction, *S. rapamycinicus* regulates the acetylation of H3K9 and H3K14 in *A. nidulans* which alters the expression of a globally acting transcriptional regulator (Fischer et al. 2018). It was also shown that the silent secondary metabolite (SM) gene cluster for orsellinic acid (*ors*) in the filamentous fungus *A. nidulans* is activated by *S. rapamycinicus* (Nutzmann et al. 2011)*.*

## Agricultural relevance of bacterial-fungal interactions

**Fig. 2:**
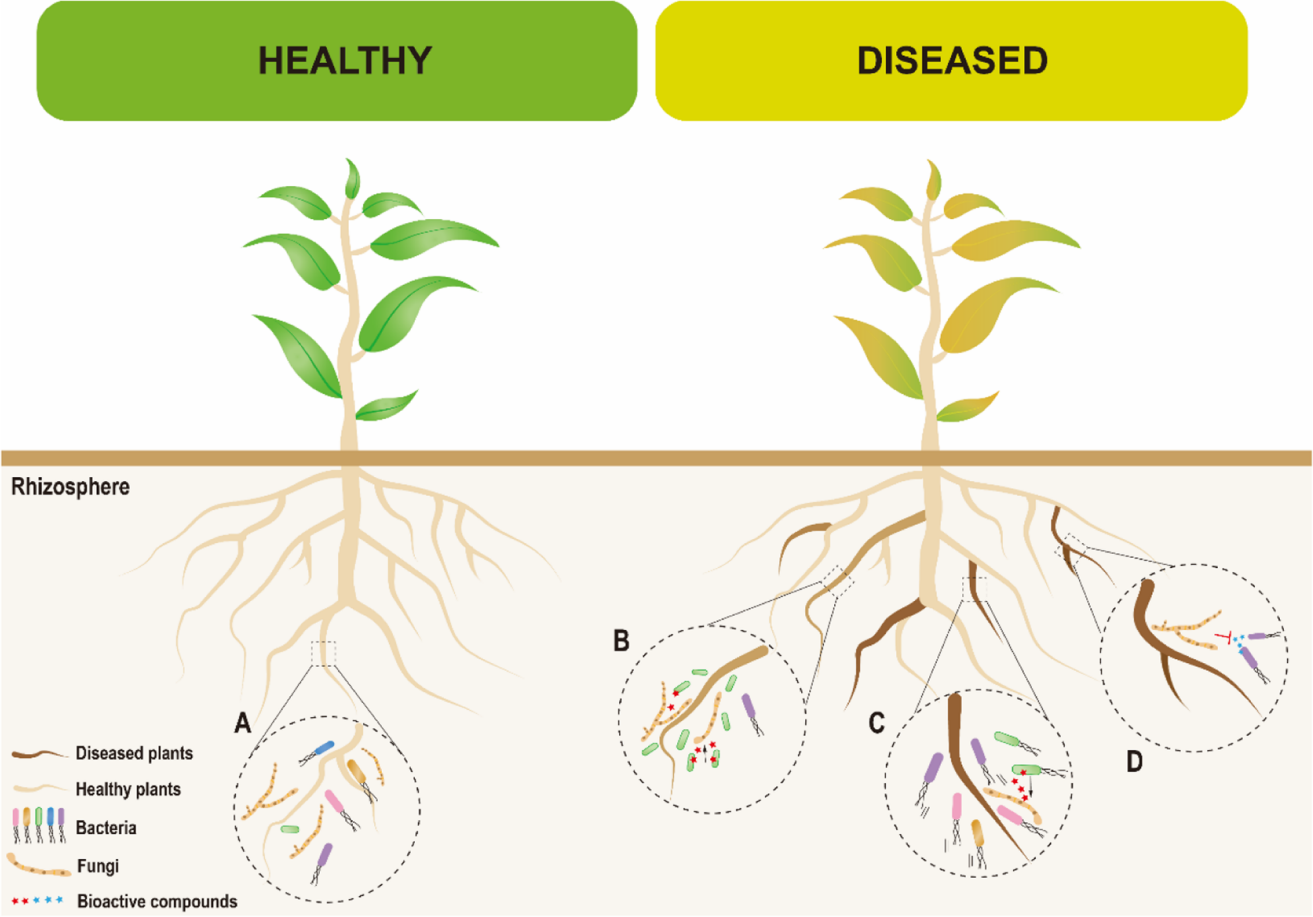
Implications of bacterial-fungal interactions (BFIs) for agriculture. **A** Bacteria can occur together with fungal pathogens in the same plant microbiota without any disease symptoms due to their regulation by BFIs. **B** Fungal pathogens can enrich antagonists by themselves which then antagonize them. **C** Infected plants can recruit beneficial bacteria via the ‘cry for help’ mechanism which then antagonize the attacking fungal pathogen. **D** Fungal pathogens commonly develop defense mechanisms when exposed to bacterial antagonism

Bacteria and fungi are widely distributed in various agricultural systems where they commonly engage in specific interactions. These interactions can influence the role of the individual interaction partners in the local environment. Currently, BFIs research in agricultural systems mainly focuses on i) the identification of ideal biocontrol strains for biological control of plant diseases, ii) the selection of suitable constituents of synthetic communities (SynComs), and iii) studying the underlying interaction mechanisms between distinct microorganisms in order to better understand fundamentals of microbial ecology. Previous research has mostly provided crucial insights into various host-protective effects of plant-associated bacteria that can efficiently antagonize fungal phytopathogens (Fig. 2).

### Exploration of BFIs in biological control applications

The control of plant diseases with biocontrol agents provides a more sustainable alternative to agrochemicals. In particular, *Bacillus, Pseudomonas, Burkholderia,* and *Trichoderma* are well-known biological control agents for controlling plant diseases. The most studied and widely exploited biological control mechanism is the deactivation of pathogens via antibiosis (Table 2). Currently, the most commonly reported bioactive compounds produced by biocontrol agents include pyoluteorin (Plt), phenazine-1-carboxylic acid (PCA), 2,4-di-acetylphloroglucinol (DAPG), pyrrolnitrin (Prn), hydrogen cyanide (HCN), and protein-type compounds (bacteriocins) which are all produced by *Pseudomonads* spp. (Deng et al. 2015; Huang et al. 2018; Trung et al. 2020) and fengycin, rapamycin and pyrrolnitrin which can be produced by *Bacillus*, *Streptomyces*, and *Burkholderia* spp. (Jung et al. 2018; Liu et al. 2005; Wang et al. 2021). So far, most biocontrol strains have been obtained mainly by isolation from plants and soil. For example, the PCN-producing biocontrol strain *P. chlororaphis* ZJU60 was isolated from the natural microbiota of wheat heads. The application of ZJU60 results in deregulation of histone acetylation and suppression of growth and virulence in *F. graminearum* (Chen et al. 2018)*.* It was also found that the biocontrol strain produced more bioactive compounds in the presence of pathogens. *Lysobacter enzymogenes* OH11 produces HSAF to inhibit the growth of pathogenic fungi; the presence of pathogens can stimulate the strain to secrete more HSAF (Qian et al. 2009; Lin et al. 2021; Qian et al. 2013).

In addition to the exploitation of antimicrobial compounds for biological control, various other mechanisms of pathogen suppression were reported. Biocontrol strains can protect plants from pathogens by changing the micro-environment. For example, *R. aquatilis* can invade the mycelium of the pathogenic fungus *F. oxysporum* that infects plant roots through pH-mediated chemotaxis (Palmieri et al. 2020). It then uses the fungal mycelium as a channel to effectively reach and colonize the roots of plants. On the other hand, acidification caused by a gluconic acid secreted by *R. aquatilis* in the rhizosphere can inhibit pathogenicity mechanisms of *F. oxysporum*, thus inhibiting infection of the host plant (Palmieri et al. 2020).

### BFI exploration for the construction of synthetic communities

Most of the currently available biocontrol studies have focused on a limited number of single microbial strains. However, microbes naturally occur in complex communities that can be influenced by their hosts, the surrounding environment, and other members of the microbial community. It was often shown that the use of different microbes in form of a defined consortium can better simulate the natural conditions (Niu et al. 2020). Usually, the construction of synthetic communities (SynComs) is based on two methods: (i) combining existing individual biocontrol bacteria based on experience and (ii) constructing microbial SynComs based on observations in natural microbiomes (Liu et al. 2019).

In theory, combinations of beneficial microorganisms to control disease should improve the biocontrol efficiency, but in practice, a combination of multiple microorganisms is sometimes not as effective as a single one. This is mainly because when beneficial bacteria are selected, the screening is conducted on the basis of their disease control effects, while microbe-microbe interactions and their compatibility are mostly ignored. Compatibility between beneficial bacteria can be explored with in vitro co-cultures or in situ *root* colonization competition tests to prove that the selected microorganisms do not inhibit each other’s growth. A successful example of constructing SynComs through experience is the combination of *Trichoderma virens* Gl006 and *Bacillus velezensis* Bs006 (Izquierdo-García et al. 2020). The lipopeptides bacillomycin D and fengycin compounds produced by *B. velezensis* inhibit filamentous fungi (Chen et al. 2007) which should negatively influence their co-application. However, Izquierdo-García et al. (2020) found that the addition of *B. velezensis* supernatant can promote the growth of *Trichoderma*. On the contrary, the addition of *Trichoderma* conidia to *B. velezensis* had no effect on the activity of *B. velezensis*, indicating a high compatibility between the two microorganisms.

Although plants have evolved their own adaptations to alleviate most biotic and abiotic stresses, they also rely on their microbial partners to survive and defend themselves against microbial invaders (Turner et al. 2013). When the plant host is attacked by pathogens, it can send out specific signals to recruit and enrich specific beneficial microorganisms, namely the plant ‘cry for help’ strategy (Yuan et al. 2018). Such observation from mechanistic microbiome studies can be harnessed for the construction of highly effective SynComs. For example, Carrion et al. (2019) found that during pathogen invasion, members of the endophytic bacteria Chitinophagaceae and Flavobacteriaceae are enriched within the plant endosphere and that they show enhanced enzymatic activities associated with fungal cell wall degradation, as well as secondary metabolite biosynthesis encoded by NRPs and PKS. They reconstructed a SynCom of *Flavobacterium* and *Chitinophaga* that provided holistic disease protection to the host plant. A simplified construction of a synthetic bacterial community through host-mediated selection was described by Niu et al. (2017). It consists of seven bacterial species (*Enterobacter cloacae, Stenotrophomonas maltophilia, Ochrobactrum pituitosum, Herbaspirillum frisingense, Pseudomonas putida, Curtobacterium pusillum, and Chryseobacterium indologenes*). This community is capable of remarkable inhibition of the phytopathogenic fungus *Fusarium verticillioides*. Under abiotic selection pressure, plants can also shape their own microbiota. A recent study analyzed the rhizosphere microbial community of garlic in different growth periods and soil types, and found that *Pseudomonas* was an important constituent in the garlic rhizosphere (Zhuang et al. 2021). Six *Pseudomonas* isolates from garlic rhizosphere were subsequently used to construct SynComs, which could promote plant growth. Overall, many SynComs were only constructed in laboratories and their field applicability remains to be confirmed in the future.

### Pathogen responses to antagonistic bacteria

Biological control studies currently mostly focus on the antagonistic microorganism that is involved in a specific interaction and on how it affects the target pathogen (mechanism, metabolites, genes, etc.). It is often ignored that fungal pathogens can also respond to antagonistic bacteria. So far, various mechanisms have been reported that can be employed by pathogens to resist bioactive compounds that are produced by bacteria. Such compounds can be deactivated by enzymatic degradation including a wide variety of substrate-specific enzymes, like acetyltransferases, hydrolases, hydratases, demethylases, and cytochrome P450-dependent monooxygenases (Morrissey and Osbourn 1999). When a sublethal concentration of 1-hydroxyphenazine produced by *Pseudomonas* sp. was added to liquid cultures of *Mycosphaerella graminicola*, the fungal catalase, peroxidase and superoxide dismutase were significantly increased, resulting in reduced oxidative stress of the fungus (Duffy et al. 2003). Pathogens can also increase their resistance by modifying targets of distinct bioactive compounds (Schisler et al. 1991). Pyochelin produced by *Burkholderia cenocepacia* 869 T2 can inhibit the growth of *Phellinus Noxius* Pn2252, the causative agent of brown root disease, in the early stage (Sahashi et al. 2012). However, after 1–2 weeks of co-cultivation of Pn2252 and 869 T2, Pn2252 developed resistance to pyochelin. MALDI-TOF IMS analysis showed that the secondary metabolism of Pn2252 changed as a response to the bioactive compound. Pn2252 can transform pyochelin and ent-pyochelin into pyochelin-Ga and ent-pyochelin-Ga, respectively; the transformed products no longer have antifungal activity (Ho et al. 2021). An interesting observation is that pathogens can also produce the corresponding SMs which represses antibiotic production. Fusaric acid, a pyridine-carboxylic acid with phyto and mycotoxigenic activity produced by *Fusarium* spp., at concentrations as low as 0.12 μg/ml repressed production of 2,4-diacetylphloroglucinol CHA0, a key factor in the biocontrol activity of *Pseudomonas fluorescens* (Duffy and Defago 1997)*.* In addition, pathogens can protect themselves by activating efflux mechanisms that prevent the accumulation of bioactive compounds inside the cell (de Waard 1997). For example, 4-diacetylphloroglucinol, phenazine-1-carboxylic acid and phenazine-1-carboxamide, broad-spectrum antibiotics produced by *Pseudomonas* spp., induced expression of multiple ABC transporter genes in *Botrytis cinerea*. Among them, phenazines were also shown to strongly induce expression of the ABC transporter gene BcatrB (Schoonbeek et al. 2002). When *Lysobacter enzymogenes* C3 and DCA, a mutant that lost inhibitory fungal activity, interacted with the rice blast pathogen, respectively, RNA-seq data showed that 100 genes were down-regulated in the wild type but up-regulated in the mutant (Mathioni et al. 2013). The up-regulated genes were hypothesized to be involved in the fungal defense response (Mathioni et al. 2013). Thus, understanding the defensive mechanisms of pathogens against antagonistic microorganisms is essential in order to improve the effect of biological control agents. It is noteworthy to mention that most studies on pathogen defense mechanisms have been almost exclusively carried out in vitro. These experiments are often not representative for in situ conditions and will require further exploration and validation in the future.

## Concluding remarks and perspectives

In nature, BFIs are highly complex and include a wide range of mechanisms where diverse molecules are involved. They enable microorganisms to recognize distinct species and to correspond with each other in a complex environment. Although various compounds involved in specific BFIs were presented, they are only derived from a small fraction of the whole microbiota that is cultivable. The majority of microbes are currently unculturable, and their potential will likely be discovered in the future by comprehensive multi-omics approaches.

In agriculture, BFIs are often explored in the frame of the development of new biocontrol strains or to explore the involved antibacterial/antifungal compounds. Currently, the application of many biocontrol agents is limited by a variety of factors, including the low survival rate and colonization ability of introduced biocontrol strains, as well as lowered expression of key antagonistic traits under field conditions. Therefore, a comprehensive understanding of the dynamic interrelationships between pathogens, antagonistic microorganisms and the environment must be included in the development of reliable biological control strategies.

The body of knowledge gained from the investigation of binary bacterial-fungal interactions will likely contribute to our understanding of complex interactions in highly diverse microbial communities. With the continuous development of microbiome research and synthetic biology, researchers will increasingly focus on the construction of synthetic communities to establish functional microbial communities as they are found in natural ecosystems. In medicine and food engineering, the Lotka-Volterra mathematical modeling approach plays a key role in guiding the design of synthetic communities (Venturelli et al. 2018). Mathematical models explain how interactions between species in natural microbial communities control community dynamics. Furthermore, it must be considered that interactions between species can significantly change with environmental conditions as well as species’ traits can change due to evolutionary adaptation (Escalante et al. 2015), which aggravates the construction of long-lasting biocontrol solutions for agriculture. A large number of scientists in theoretical ecology and evolution are working on the exploration of the stability of interactions between species in communities and the process of evolution, which can provide information for modeling and obtaining evolutionarily stable communities (Nowak et al. 2004). Therefore, combined insights into BFIs from microbial ecology, metagenomics and other meta-omics approaches, as well as big data analysis, mathematical model construction, microfluidic and other technologies to construct SynComs is the future development trend.

## Data Availability

Not applicable.

## Abbreviations

BFIs: bacterial-fungal interactions
SMs: secondary metabolites
VOCs: volatile organic compounds
QS: Quorum-sensing
AMF: arbuscular mycorrhizal fungi
*Ca*gg: *Candidatus* Glomeribacter gigasporarum
MRE: *Mollicutes*-related endobacteria
T2SS: the type II protein secretion system
EPS: extracellular polysaccharides
DMDS: dimethyl disulfide
MI: methyl isovalerate
AHL: *N*-acyl-L-homoserine lactone
PQS: *Pseudomonas* quinolone signal
BGCs: biosynthetic gene clusters
PCN: phenazine-1-carboxamide
GlcA: gluconic acid
Plt: pyoluteorin
PCA: phenazine-1-carboxylic acid
DAPG: 2,4-di-acetylphloroglucinol
Prn: pyrrolnitrin
HCN: hydrogen cyanide
HSAF: heat-stable antifungal factor
SynComs: synthetic communities
